# Genomic resources of aquatic Lepidoptera, *Elophila obliteralis* and *Hyposmocoma kahamanoa*, reveal similarities with Trichoptera in amino acid composition of major silk genes

**DOI:** 10.1093/g3journal/jkae093

**Published:** 2024-05-09

**Authors:** Jacqueline Heckenhauer, David Plotkin, Jose I Martinez, Jacob Bethin, Steffen U Pauls, Paul B Frandsen, Akito Y Kawahara

**Affiliations:** Senckenberg Research Institute and Natural History Museum Frankfurt, Terrestrial Zoology, 60325 Frankfurt am Main, Germany; LOEWE Centre for Translational Biodiversity Genomics (LOEWE-TBG), 60325 Frankfurt am Main, Germany; McGuire Center for Lepidoptera and Biodiversity, Florida Museum of Natural History, University of Florida, Gainesville, FL 32611, USA; McGuire Center for Lepidoptera and Biodiversity, Florida Museum of Natural History, University of Florida, Gainesville, FL 32611, USA; McGuire Center for Lepidoptera and Biodiversity, Florida Museum of Natural History, University of Florida, Gainesville, FL 32611, USA; Senckenberg Research Institute and Natural History Museum Frankfurt, Terrestrial Zoology, 60325 Frankfurt am Main, Germany; LOEWE Centre for Translational Biodiversity Genomics (LOEWE-TBG), 60325 Frankfurt am Main, Germany; Institute for Insect Biotechnology, Justus-Liebig-University, 35392 Gießen, Germany; Department of Plant and Wildlife Science, Brigham Young University, Provo, UT 84602, USA; Data Science Lab, Smithsonian Institution, Washington, DC 20560, USA; McGuire Center for Lepidoptera and Biodiversity, Florida Museum of Natural History, University of Florida, Gainesville, FL 32611, USA

**Keywords:** aquatic moth, *Elophila obliteralis*, h-fibroin, *Hyposmocoma kahamanoa*, silk gene, underwater silk

## Abstract

While most species of butterflies and moths (Lepidoptera) have entirely terrestrial life histories, ∼0.5% of the described species are known to have an aquatic larval stage. Larvae of aquatic Lepidoptera are similar to caddisflies (Trichoptera) in that they use silk to anchor themselves to underwater substrates or to build protective cases. However, the physical properties and genetic elements of silks in aquatic Lepidoptera remain unstudied, as most research on lepidopteran silk has focused on the commercially important silkworm, *Bombyx mori*. Here, we provide high-quality PacBio HiFi genome assemblies of 2 distantly-related aquatic Lepidoptera species [*Elophila obliteralis* (Pyraloidea: Crambidae) and *Hyposmocoma kahamanoa* (Gelechioidea: Cosmopterigidae)]. As a step toward understanding the evolution of underwater silk in aquatic Lepidoptera, we used the genome assemblies and compared them to published genetic data of aquatic and terrestrial Lepidoptera. Sequences of the primary silk protein, h-fibroin, in aquatic moths have conserved termini and share a basic motif structure with terrestrial Lepidoptera. However, these sequences were similar to aquatic Trichoptera in that the percentage of positively and negatively charged amino acids was much higher than in terrestrial Lepidoptera, indicating a possible adaptation of silks to aquatic environments.

## Introduction

With over 160,000 described species, butterflies and moths (Lepidoptera) are a hyperdiverse terrestrial insect lineage (Nieukerken *et al.* 2011). Interestingly, 0.5% of described species are known to have an unusual aquatic larval stage (Pabis 2018), similar to caddisflies (Trichoptera), the primarily aquatic sister order of Lepidoptera within superorder Amphiesmenoptera. Trichoptera and Lepidoptera diverged from a silk-spinning ancestor around 290 million years ago (Misof *et al.* 2014). The aquatic trichopteran larvae are known to produce unique silks used to create structures that allow them to survive underwater. The genetic basis of silks in aquatic Lepidoptera has not been examined, as most studies on lepidopteran silk have focused on the commercially important silkworm, *Bombyx mori* (Tsujimoto and Suzuki 1979; Mita *et al.* 1994; Tanaka *et al.* 1999; Zhou *et al.* 2000; Inoue *et al.* 2000; Tong *et al.* 2022). Similar to caddisflies, aquatic lepidopteran larvae also anchor themselves to substrates underwater and several species use silk to build protective cases from various organic or mineral material during larval and pupal stages (Mackay and Wiggins 1979). Given that Trichoptera and aquatic Lepidoptera transitioned into freshwater environments independently, this gives us an opportunity to evaluate whether there are shared convergent adaptations in the molecular structure of underwater silk. A first step to address this question is sequencing the major silk gene, *h-fibroin*, as it is one of the primary genes associated with silk structure and function (Zhou *et al.* 2000). *H-fibroin* is long (>15 kilobase pairs) and highly repetitive. With new long-read sequencing methods, the repetitive regions can be sequenced successfully (Kawahara *et al.* 2022).

In this study, we generated PacBio HiFi genome assemblies for 2 aquatic Lepidoptera species [*Elophila obliteralis* (Pyraloidea: Crambidae) and *Hyposmocoma kahamanoa* (Gelechioidea: Cosmopterigidae)]. These 2 species are nested within families whose larvae are predominantly terrestrial, thus indicating independent transitions to the aquatic habitat within clades that are separated by more than 100 million years of evolution (Kawahara *et al.* 2019). *Elophila obliteralis* is distributed throughout eastern North America ranging from Florida to as far north as Manitoba, Canada, and as far west as Texas, and has been introduced to England, Hawaii, and British Columbia (Dyar 1906). The larva of *E. obliteralis* feeds on aquatic plants with a host plant breadth of more than 60 species (Habeck and Cuda 2009) and the moth has been used to control invasive aquatic plants (e.g. *Nymphoides peltata* ). The larva of *E. obliteralis* creates a case by using silk to hold together cut-up leaf pieces. A young instar larva creates water-filled cases and anchors itself to their host plant with silk; an older instar will build a case that contains an air pocket and is free-floating until pupation when the larva reattaches its case to the host plant and begins to spin a silk cocoon. After emergence and mating, the female lays eggs on the margins of the leaves of aquatic plants (Dyar 1906). *Hyposmocoma* contains more than 400 species (Dupont and Rubinoff 2015), all endemic to the Hawaiian Islands (Schmitz and Rubinoff 2011; Kawahara and Rubinoff 2013; Haines *et al.* 2014). Species of this genus are ecologically diverse and build a diverse set of cases used as shelters. Recently, in this genus, 11 aquatic species were discovered in multiple, unique lineages in areas with flowing freshwater across the Hawaiian Islands (Schmitz and Rubinoff 2011; Medeiros *et al.* 2017). The larva of *H. kahamanoa* is found on porous volcanic rocks in streams, both above and underwater. When submerged, it uses a strand of silk to keep itself attached to substrate. Although many aquatic lepidopteran larvae maintain a bubble of air inside of their cases, this is rare in *H. kahamanoa*; it is believed that all members of the genus *Hyposmocoma* instead respire via direct diffusion of oxygen through their hydrophilic skin (Schmitz and Rubinoff 2011). The larva has been recorded to feed mainly on fresh and dried algae, and sometimes on lichens. *H. kahamanoa* is multivoltine with multiple annual broods. Larval cases of *H. kahamanoa* have a cone-shaped structure and consist of sand and pebbles woven together with silk filaments (Schmitz and Rubinoff 2011), superficially resembling caddisfly cases.

We used the genomic assemblies generated in this study, in addition to 3 publicly available high-quality genomes of aquatic Lepidoptera, to identify h-fibroin sequences and characterize their primary structure. To examine differences between terrestrial and aquatic silk usage, we compared the amino acid composition of the h-fibroins of aquatic Lepidoptera to that of 11 species of Trichoptera, as well as to 5 representatives of terrestrial Lepidoptera (see Heckenhauer *et al.* 2023).

Aquatic insects are under-represented in genomic research (Hotaling *et al.* 2020). Our study provides much-needed genomic data to enable future studies on the genomic basis of the evolution of underwater silk. Furthermore, these genomes may contribute to future comparative genomic studies on understanding how insects that are typically terrestrial have adapted to aquatic environments.

## Materials and methods

### Sampling strategy, sample preparation details, and sequencing methods

A single individual of each species was wild-caught as a final instar larva. *E. obliteralis* was collected in an artificial pond on the University of Florida campus in Gainesville, Florida, USA (29.636428, −82.370764). *H. kahamanoa* was collected at Manoa Stream on Oahu Island, Hawaii, USA (21.333115, −157.800039). High molecular weight genomic DNA was extracted from the thorax of *E. obliteralis* using the Qiagen genomic tip DNA extraction kit. High molecular weight DNA was sheared to 18 kbp with a Diagenode Megaruptor, and the BluePippin system (Sage Science, Beverly, MA, USA) was used to collect fractions containing >15-kbp fragments for library preparation. Genomic libraries were prepared following the SMRTbell Express Template Prep. Kit 2.0 protocol (PacBio, Menlo Park, CA, USA). The library was sequenced on two 8M SMRTcells with 30-hour movie times in ccs (circular consensus sequencing) mode using the PacBio Sequel II system.

For *H. kahamanoa*, high molecular genomic DNA was extracted from the head and thorax using an Agilent DNA extraction kit and sequencing libraries were prepared using the SMRTbell Express Template Prep. Kit 2.0 (PacBio, Menlo Park, CA, USA) following the ultra-low protocol. The library was sequenced on a single 8M SMRTcell in ccs mode with a 30-hour movie time using the PacBio Sequel II system.

DNA extractions, library preparation, and sequencing were performed at the DNA Sequencing Center at Brigham Young University (Provo, UT, USA).

### Raw data processing

HiFi reads (reads with quality above Q20) were computed from the raw data using PacBio SMRTlink software. DeepConsensus v.1.0.0 (Baid *et al.* 2023) was used to generate additional HiFi reads from subreads following instructions at https://github.com/google/deepconsensus. For this, to generate a draft consensus, pbccs 6.4.0 *ccs* was run with the --min-rq=0.88 flag. Next, subreads were aligned to the draft consensus sequence using actc (https://github.com/PacificBiosciences/actc). Then, DeepConsensus was used to generate polished reads by using gap-aware sequence transformers to correct errors in the ccs reads. Since the ultra-low protocol used for library prep of the *H. kahamanoa* DNA involved a whole-genome amplification step, pbmarkdup --rmdup (https://github.com/PacificBiosciences/pbmarkdup) was used to remove duplicates.

### Genome assembly and quality assessment

The ccs reads from the previous steps were assembled into contigs using HiFiASM v0.13-r307 (Cheng *et al.* 2021). HiFiASM generates a primary and an alternate assembly. MitoHiFi v3.2 (Uliano-Silva *et al.* 2023) was run on the primary assemblies using the most closely related complete mitogenomes (*Elophila interruptalis* KC894961 and *Meleonoma mirabilis* MW366996). Eleven potential mitogenome contigs (25,500–41,908 bp) detected by MitoHiFi were excluded from the *E. obliteralis* assembly. One potential mitogenome contig was excluded from the *H. kahamanoa a*ssembly. The resulting nuclear genome assemblies were screened for potential contaminations with taxon-annotated GC-coverage (TAGC) plots using BlobTools v1.1.1 (Laetsch and Blaxter 2017) as described in Heckenhauer *et al.* (2023). For the *E. obliteralis* assembly, contigs not assigned to Arthropoda that were not in the range of the coverage (4.6591–103.4052) and GC content (0.3314–0.5401, see Supplementary Fig. 1) of the arthropod-assigned contigs were filtered out using SAMtools v1.13 faidx (Li *et al.* 2009). In addition, NCBI detected *Wolbachia* endosymbionts in 2 contigs. These were subsequently trimmed from the assembly. For the *H. kahamanoa* assembly, contigs not assigned to Arthropoda that were not in the range of the coverage (0.3329–131.0185) and GC content (0.3278–0.5977, see Supplementary Fig. 2) of the arthropod-assigned contigs were filtered out using SAMtools v1.13 faidx. In addition, NCBI detected a vector in 1 contig, which was trimmed from the assembly. The assembly continuity was evaluated using QUAST v5.0.2 (Gurevich *et al.* 2013) and completeness was calculated with BUSCO v5.2.2 (Simão *et al.* 2015; Waterhouse *et al.* 2018) using the lineage dataset endopterygota_odb10 in genome mode, as well as with compleasm (Huang and Li 2023) using -l endopterygota. The back-mapping rate of the HiFi reads to the assemblies was calculated using backmap.pl v0.5 (Schell *et al.* 2017; Pfenninger *et al.* 2022) with the parameter -hifi as described in Heckenhauer *et al.* (2023).

### Genome-size estimation and genome profiling

Genome size was estimated using sequencing reads and a *k*-mer-based statistical approach using JELLYFISH v2.2.10 (Marçais and Kingsford 2011) and Genomescope2 (Ranallo-Benavidez *et al.* 2020) as described in Heckenhauer *et al.* (2023). In addition, we used backmap.pl to obtain a second genome-size estimate based on the mapped nucleotides divided by mode of the coverage distribution (>0) as described in Schell *et al.* (2017) and Pfenninger *et al.* (2022).

### Structural and functional genome annotation

Repetitive elements were identified and classified de novo with RepeatModeler2 (Flynn *et al.* 2020) in the genome assemblies and masked using RepeatMasker (http://www.repeatmasker.org) following Heckenhauer *et al.* (2019). Repeatmodeler libraries and stk files were uploaded to Dfam (Wheeler *et al.* 2013; Hubley *et al.* 2016; Storer *et al.* 2021). Structural annotations were conducted with Braker3 (Lomsadze *et al.* 2005; Stanke *et al.* 2006, 2008; Gotoh 2008; Iwata and Gotoh 2012; Buchfink *et al.* 2015; Hoff *et al.* 2016, 2019; Brůna *et al.* 2020) with the pre-partitioned OrthoDB v.11 Arthropoda clade (available at: https://bioinf.uni-greifswald.de/bioinf/partitioned_odb11/Arthropoda.fa.gz). Completeness of the annotation was assessed with BUSCO in protein mode. Ratio of mono- to multiexonic genes was calculated with the python script analyze_exons.py from GALBA (Brůna *et al*. 2023: https://raw.githubusercontent.com/Gaius-Augustus/GALBA/master/scripts/analyze_exons.py). For functional annotations of the predicted proteins, we used BlastP to search against the ncbi-blast protein database (Altschul *et al.* 1990) with an *e*-value cutoff of 10^−4^ and –max_target_seqs set to 10. Functional annotation and GO terms were assigned with the command line version of Blast2GO v.1.4.4 (Conesa and Götz 2008).

### Identification and annotation of heavy-chain fibroins

Three high-quality aquatic Lepidoptera assemblies (*Acentria ephemerella* (Boyes and Mulhair 2024a), *Nymphula nitidulata* (Boyes and Mulhair 2024b), *Parapoynx stratiotata* (Boyes and Chadd 2022)) were published by the Wellcome Sanger Institute as part of the Darwin Tree of Life Project (The Darwin Tree of Life Consortium 2022, see https://www.darwintreeoflife.org/). *H-fibroin* genes were identified in the published and newly produced primary and alternate assemblies by using tBLASTn to search the assemblies with the conserved n- and c-termini of *Plodia interpunctella* (Kawahara *et al.* 2022) in Geneious Prime 2022.1.1 (https://www.geneious.com, see Supplementary Table 2) with default settings. After verifying that both BLAST hits (n- and c-terminus hits) were located on the same contig in the genome assembly, the sequences and 5,000 bp of flanking regions were extracted from the assembly using the sequence view “extract” in Geneious and this region was annotated using Augustus v.3.3.3 (Stanke *et al.* 2008). Introns that did not disrupt reading frames were manually removed from the annotation. Protein-coding nucleotide sequences were translated with the Geneious Tool “Translate” using the standard genetic code. Signal peptides were predicted with the SignalP 6.0 server (Teufel *et al.* 2022) using the following settings: organism = Eukarya, model mode = slow.

### Comparison of heavy-chain fibroin sequences

To visualize the differences in repeat structure among species, a custom visualization script was used to split the gene into repeat modules (https://github.com/AshlynPowell/silk-gene-visualization/tree/main). ExPASy ProtParam (https://web.expasy.org/protparam/) was used to compute the molecular weight and the amino acid composition of each sequence. To compare conserved regions of the h-fibroin proteins, we aligned the n- and c-termini each (without the signal peptide) in Geneious using the Muscle 3.8.425 (Edgar 2004) plugin with a maximum of 1,000 iterations. We compared % pairwise identity and % of identical sites in Geneious. We conducted alignments with terminal regions of previously published h-fibroin sequences of Trichoptera and terrestrial Lepidoptera and calculated phylogenetic trees with RAxML 8.2.11 (Stamatakis 2014) with protein model GAMMA GTR and the rapid bootstrapping algorithm with 1,000 bootstrap replicates. Consensus trees were generated with the Geneious Consensus Tree Builder with a support threshold of 50% (see Supplementary Figs. 8 and 10).

## Results and discussion

### Description of the genomes—sequencing coverage and quality of genome assembly

Whole-genome assemblies were generated for *E. obliteralis* and *H*. *kahamanoa* using PacBio HiFi sequencing in addition to previously published high-quality genome assemblies of aquatic Lepidoptera (Pyraloidea: Crambidae: *A. ephemerella*, *N. nitidulata*, *P. stratiotata*) and 1 medium-quality assembly of *H. kahamanoa*. The genomic resources generated here represent a step toward abating the under-representation of aquatic insects in genomic research (Hotaling *et al.* 2020).

For *E. obliteralis*, sequencing resulted in 2,239,568 HiFi reads (total 32.56 Gbp). These numbers were increased to 2,395,440 reads (34.91 Gbp, approx. 50× coverage) after running DeepConsensus. Genomescope2 estimated a genome size of 590.74 Mbp with 74.8% unique sequence (see Supplementary Fig. 3). Backmap.pl estimated a genome size of 628.33 Mbp (see Supplementary Fig. 4). The contamination-free assembly was of high quality with respect to contiguity and BUSCO gene completeness. The assembly length was 646,136,679 bp consisting of 103 contigs with a contig N50 of 23,093,419. BUSCO analysis recovered 99% complete orthologs of which 98.8% were single copy. Compleasm recovered an ortholog completeness of 99.67% (99.53% single, 0.14% duplicated). Re-mapping the reads to the assembly revealed that 34.56 Gbp (99%) could be unambiguously placed with the expected coverage distribution per position.

For *H. kahamanoa*, 2,859,904 reads (33.87 Gbp, approx. 42× coverage) were obtained after running DeepConsensus and pbmarkdups. Genomescope2 estimated a genome size of 590,740,933 Mbp with 70.9% unique sequence (see Supplementary Fig. 5). However, Backmap.pl estimated a genome size of 827.25 Mbp (see Supplementary Fig. 6) which is in line with the length of the contamination-free assembly (803,821,239 bp). Since repetitive elements may affect *k*-mer estimates (Austin *et al.* 2017; Sánchez-Herrero *et al.* 2019), they likely account for the discrepancy of the Genomescope2 genome estimate compared to Backmapl.pl results and assembly length. Compared to other aquatic Lepidoptera, the *H. kahamanoa* assembly was of lower quality as assessed via contiguity and BUSCO. This is likely because the PacBio ultralow library preparation includes a whole-genome amplification step, which can introduce errors in species with genome sizes larger than 400 Mbp. However, despite this, the assembly contiguity was better than that of a previously published *H. kahamanoa* assembly GCA_003589595.1 (Table 1). BUSCO analysis recovered 89.8% complete orthologs of which 81.8% were single copy. Compleasm recovered an ortholog completeness of 90.15% (82.63% single, 7.53% duplicated). Re-mapping the reads to the assembly revealed that 33.09 Gb (97.7%) could be unambiguously placed with expected coverage distribution per position.

**Table 1.** Comparison of currently published genome assemblies of aquatic Lepidoptera.

Species	Superfamily/family (silk use)	Accession number	Assembly length (Mbp)	N50 (contig/scaffold, Mbp)	No. of contigs/scaffolds)	BUSCO (%, n = 2,124)/ compleasm (%)
*Acentria ephemerella*	Pyraloidea/Crambidae	GCA_943193645.1	340.8	12.3/12.3	37/35	C:99.3[S:99.1,D:0.2],F:0.2,M:0.5/C:99.67[S:99.62,D:0.05],F:0.28,M:0.05
*Elophila obliteralis* ^a^	Pyraloidea/Crambidae	JAVLVO000000000	646.1	23.1/na	103/na	C:99.0[S:98.8,D:0.2],F:0.4,M:0.6/ C:99.67[S:99.53,D:0.14],F:0.24,M:0.09
*Hyposmocoma kahamanoa*	Gelechioidea/Cosmopterigidae	GCA_003589595.1	731.4	0.059/13.5	18,849/2,929	C:93.0[S:91.9,D:1.1],F:3.2,M:3.8/ C:99.67[S:99.53,D:0.14],F:0.24,M:0.09
*Hyposmocoma kahamanoa* ^a^	Gelechioidea/Cosmopterigidae	JAVLVP000000000	803.8	0.41/na	4,200/na	C:89.8[S:81.8,D:8.0],F:4.7,M:5.5/ C:90.15[S:82.63,D:7.53],F:5.37,M:4.47
*Nymphula nitidulata*	Pyraloidea/Crambidae	GCA_947347705.1	635.8	21.2/22.2	45/40	C:99.4[S:99.1,D:0.3],F:0.2,M:0.4/ C:99.72[S:99.58,D:0.14],F:0.24,M:0.5
*Parapoynx stratiotata*	PyraloideaCrambidae	GCA_910589355.1	478.2	13.8/17.1	62/32	C:98.8[S:98.4,D:0.4],F:0.7,M:0.5/ C:99.63[S:99.44,D:0.19],F:0.33,M:0.05

Percentage of complete BUSCOs is given based on BUSCO 5.2.2 and compleasm using the endopterygota_odb10 dataset.

C, complete; S, single; D, duplicated; F, fragmented; M, missing.

^a^This study.

### Structural and functional genome annotation

Both genomes had comparable repeat compositions. A total of 52.70% of the *E. obliteralis* genome was classified as repetitive (54.2% interspersed repeats). More than half of the interspersed repeats, 29.87%, could not be classified by comparison with known repeat databases and therefore may be specific for Lepidoptera. A total of 58.75% of the *H. kahamanoa* genome assembly was masked as repeats. A total of 56.09% of the annotated repeats were interspersed repeats and 25.32% of repeats remained unclassified. Details on the repeat classes are given in Supplementary Table 1. Structural annotations resulted in the prediction of 21,179 and 35,668 proteins in *E. obliteralis* and *H. kahamanoa*, respectively. BUSCO analysis revealed ortholog completeness of 95.9 and 87% for the *E. obliteralis* and *H. kahamanoa* annotations, respectively (see Supplementary Table 3). Further descriptive statistics are given in Supplementary Table 3.

Of the annotated proteins, for *E. obliteralis*, 22.24% returned significant sequence alignments but could not be linked to gene ontology entries, 12.46% were mapped to Gene Ontology (GO) terms, and 58.58% were functionally annotated with Blast2GO. Only 6.63% did not have significant BLAST hits. For *H. kahamanoa*, functional labels were assigned to 47.43% of the annotated proteins, 29.65% returned significant sequence alignments but could not be linked to any GO entries. A total of 18.62% of the GO mapped dataset did not obtain an annotation assignment. A total of 4.3% were analyzed with BLAST but failed to obtain significant hits (see figshare: https://doi.org/10.6084/m9.figshare.24547678.v1). The major biological processes found in the 2 genomes were cellular, followed by metabolic processes. Binding and catalytic activity were the largest subcategories in molecular function. Regarding the cellular component category, most genes were assigned to the cellular anatomical entity and protein-containing complex.

### Characterization of allele length, primary structure, and amino acid composition of the h-fibroin

**Fig. 1.**
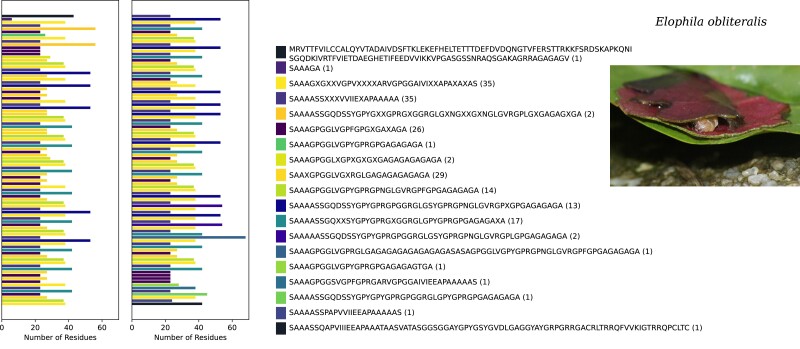
Schematic representation of the h-fibroin of *Elophila obliteralis*. The gene is split into 2 panels, starting in the left panel and continuing in the right panel. Identity and ordering of repeat motifs are shown. Repetitive units with the n-terminus and transfer region at the beginning and the c-terminus and transfer region at the end are shown as black bars. “X” indicates a variable site. The legend with the ordering of the repeats is shown on the left. The numbers in parentheses refer to the number of times that particular motif is repeated across the gene.

**Fig. 2.**
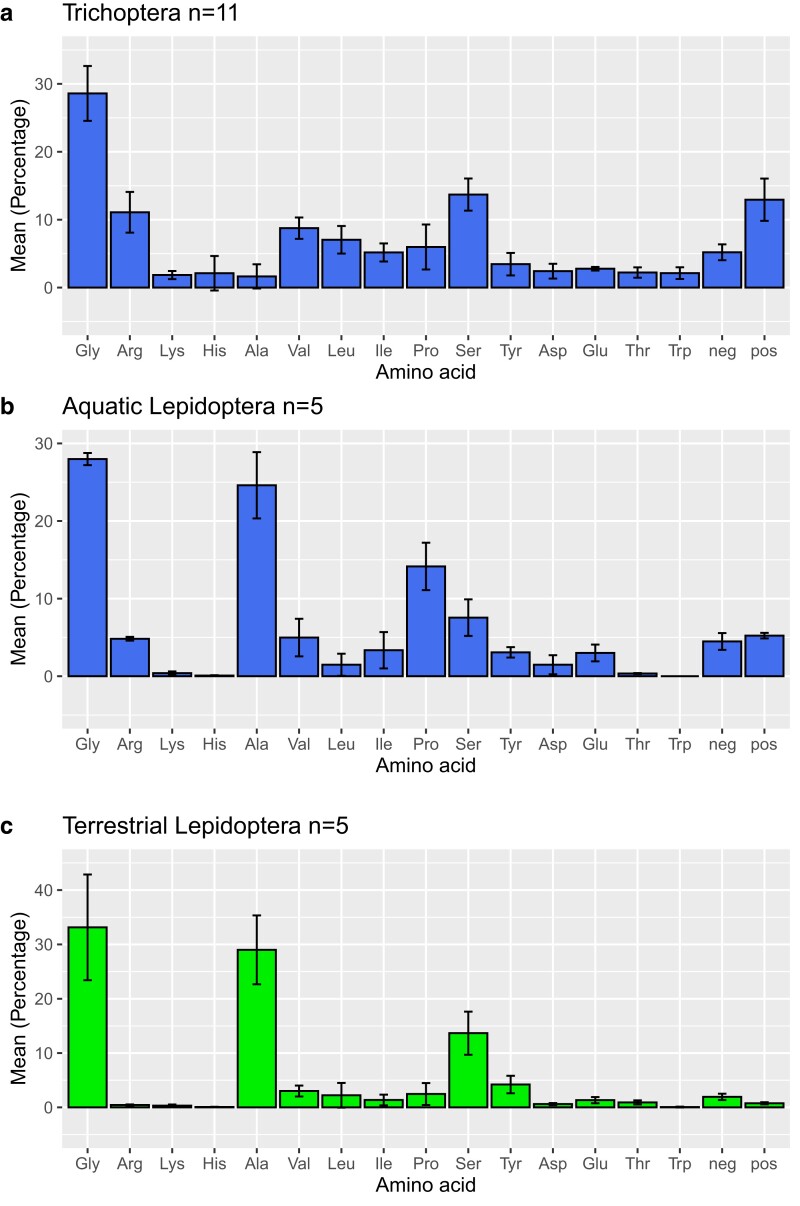
Mean amino acid composition (%) of full-length h-fibroins of Trichoptera (a) and aquatic (b) /terrestrial (c) Lepidoptera. n = number of species investigated.

H-fibroin encodes the major component of silk proteins. In this study, gene and protein sequences were identified in the *E. obliteralis* assembly, as well as in 3 previously published genome assemblies of aquatic Lepidoptera (Table 2, Fig. 1, see Supplementary Figs. 11–13). Unfortunately, we were unable to recover the full-length *h-fibroin* from *H. kahamanoa*. However, with the newly sequenced assembly in this study, the full c-terminus as well as partial n-terminus and parts of the repetitive regions were obtained (see Supplementary Note 1). The identified *h-fibroin* sequences confirm previous findings of allelic variation silk genes (Frandsen *et al.* 2023). Heterozygosity of the full-length *h-fibroin* alleles was detected in all 4 species (Table 2). Allele length differences were <5%, except for *N. nitidulata* (>10%, Table 2). The full-length *h-fibroin* genes of the of the 4 species had a similar organization of introns and exons characterized by a short exon (42 bp) and a long exon (16,650–21,516 bp) which are separated a single intron (159–1,001 bp), leading to a total length of 17,693–21,994 bp. The non-repetitive n- and c-termini were highly conserved across the investigated species, as well as with the terrestrial Lepidoptera which were used for a comparison (see Supplementary Figs. 7 and 9). The n-terminus contained 87 residues without the signal peptide. There were 63.2% identical sites (% of columns in the alignment where all sequences are identical) between the 5 species and 83.9% between the 4 Pyraloidea. Pairwise identity (% of pairwise residues identical in the alignment, including gap vs non-gap residues, but excluding gap vs gap residues) was 81.4% among all species, 91.6% among Pyraloidea. The c-terminus comprised 40 residues with 72% (Pyraloidea: 72.5%) identical sites and 72% (Pyraloidea: 76.7%) pairwise identity. A conserved cysteine was detected at position 19 in the c-terminus alignment (see Supplementary Fig. 9), which is likely functionally important. In the silkworm, *B. mori*, the terminal cysteine of the c-terminus forms an intermolecular disulfide bond with the light chain fibroin and is thus vital for the structure, stability, and secretion of the fibroin complex (Tanaka *et al.* 1999; Stewart *et al.* 2022). The terminal domains flanked a central region, composed entirely of repeating sequence blocks. Similar to terrestrial Lepidoptera, the repeating sequence blocks contain characteristic amino acid repeats, polyalanine and poly(glycine-alanine) domains which form the semicrystalline protein structure. The glycine- and alanine-rich regions are interspersed with amorphous motifs (Fig. 1, see Supplementary Figs. 14–18). The large, repetitive central domain of the h-fibroin is responsible for the properties of silk (Malay *et al.* 2016; Guo *et al.* 2018; Kono *et al.* 2019). Moreover, non-essential amino acids are the dominant residues in insect silk genes, to prevent limitations by protein content in insect diets (Sutherland *et al.* 2010). However, distinct patterns of amino acid compositions were observed between aquatic and terrestrial Lepidoptera. Similar to Trichoptera, a higher percentage of positively and negatively charged amino acids was detected compared to terrestrial Lepidoptera (Fig. 2). Negatively charged amino acids (aspartic acid and glutamic acid) ranged from 3.2–5.8% (mean = 4.48%, stabw, = 1.08) in the full-length h-fibroin of aquatic Lepidoptera (Supplementary Figs. 14–18) and 4–7.7% (mean = 5.2%, stabw. = 1.17) in Trichoptera h-fibroin (Heckenhauer *et al.* 2023) but only 1.1–2.4% (mean = 1.94%, stabw. = 0.58) in terrestrial Lepidoptera h-fibroin (Kono *et al.* 2019; Heckenhauer *et al.* 2023) (Fig. 2). Positively charged amino acids (arginine, lysine) summed up to 4.7–5.4% (mean = 5.23%, stabw. = 0.36) in aquatic Lepidoptera full-length h-fibroins but were much lower in terrestrial Lepidoptera [0.5–1.02%, (mean = 0.76%, stabw. = 0.2)] and again much higher in Trichoptera (7.6–16.9% (Heckenhauer *et al.* 2023), mean = 12.94%, stabw. = 3.11, see data file S1 at figshare: https://doi.org/10.6084/m9.figshare.24547678.v1). A study on the black fly *Simulium vittatum* (Diptera), whose larva are also aquatic, similarly showed higher abundance of charged amino acids in the central core of the silk gene, and hypothesized that this maybe an adaptation for aquatic silks because, in an aquatic environment, hydrophobicity could lead to clumping (Papanicolaou *et al.* 2013). The greater proportion of charged amino acids might instead lead to proteins that are less hydrophobic compared to terrestrial silks (Papanicolaou *et al.* 2013). In addition to differences in amino acid composition when compared with terrestrial silks, the silk proteins of aquatic Trichoptera have been shown to be phosphorylated (Stewart and Wang 2010) to increase their hydrophilic properties. Future studies should investigate if this is also the case for silk proteins of aquatic Lepidoptera. A higher proline content was observed in the full-length h-fibroin of the aquatic Lepidoptera ranging from 11.6 to 18.6% (mean = 14.15%, stabw. = 3.06). A higher % of proline was also observed in capture net-/retreat-making caddisflies (mean: 10.8%, stabw. = 1.09 (Heckenhauer *et al.* 2023)). This was hypothesized to be linked to the mechanical properties of the silk, such as a potentially enhanced extensibility of the silk fiber, important to prevent breaking when capturing prey in these nets. The investigated species in this study use silk for various purposes. Larvae of *P. stratiotata* build tube like cases made by spinning threads between leaves of aquatic plants (Vallenduuk and Cuppen 2004). Larvae of *N. nitidulata* build cases consisting of 2 parts of leaves and pupate in a cocoon that is tied on water plants (Vallenduuk and Cuppen 2004). In *A. ephemerella*, larvae live in silken nets they spin around their foodplants and pupation takes place in cocoons under the water surface (Vallenduuk and Cuppen 2004; Kriska 2023). Larvae of *Elophila* make cases out of cut-up pieces of leaves and silk. These varying uses of silks might require different mechanical properties compared to the silk of terrestrial Lepidoptera. Thus, mechanical testing of the aquatic silk is important to generate and test hypotheses on the higher percentage of proline in these species. Moreover, there are several other aquatic lineages of Lepidoptera and future studies including these should confirm the patterns found in this study. However, the data presented here serve as the first step to studying underwater adhesive silk evolution in aquatic Lepidoptera.

**Table 2.** Full-length *h-fibroins* of 12 caddisfly species derived from long-read sequencing ordered by silk usage.

Species	Allele	Gene length	CDS	Exon 1	Intron 1	Exon 2	Protein size	Molecular weight (kD)
*Acentria ephemerella*	pri.	19,450	18,456	42	994	18,414	6,151	535.1
	alt.	20,467	19,473	42	994	19,431	6,490	565.2
*Elophila obliteralis* ^a^	pri.	18,651	18,492	42	159	18,450	6,163	528.8
	alt.	19,479	19,320	42	159	19,278	6,439	545.4
*Nymphula nitidulata*	pri.	17,693	16,692	42	1,001	16,650	4,563	494.5
	alt.	19,706	18,705	42	1,001	18,666	6,233	553.7
*Parapoynx stratiotata*	pri.	21,994	21,558	42	436	21,516	7,185	614.6
	alt.	21,475	21,054	42	421	21,012	7,017	600.7

pri., primary assembly; alt., alternate assembly; CDS = protein-coding DNA; gene length, CDS, exon, and intron in bp; protein size in amino acids.

^a^This study.

## Conclusion

Here, we use high-quality genome assemblies of aquatic Lepidoptera to compare major silk genes. Similar to those of caddisflies, the percentage of positively and negatively charged amino acids was higher compared to those of terrestrial Lepidoptera indicating a possible adaptation of silks to aquatic environments.

Further, the genome assemblies provide in this study are key resources for future genomic research in the field of insect evolution, especially for examination of the genomic basis of adaptations to freshwater allowing transitions from terrestrial to aquatic environments. For example, the genomes could be used to identify genomic features that are associated with the transition to an aquatic life history using comparative genomic analysis, such as genome-wide searches for signatures of selection, conservation, and structural variation in genes important for vision, thermal tolerance, respiratory, olfactory, and metabolic processes.

## Supplementary Material

jkae093_Supplementary_Data

## Data Availability

Raw sequence data (SRR25936165, SRR25936466), genome assemblies (JAVLVO000000000, JAVLVP000000000), and sample information (SAMN35814731, SAMN35814732) are all available from NCBI under Bioprojects PRJNA985887 (*Elophila obliteralis*) and PRJNA985888 (*Hyposmocoma kahamanoa*). H-fibroin sequences are available at GenBank under the following accession numbers: *Elophila obliteralis*: OR533279 (primary allele), OR533280 (alternate allele), *Hyposmocoma kahamanoa*: OR533281 (partial n-term), and OR533282 (partial c-term). H-fibroin sequence data obtained from previously published genome assemblies are available in the Third Party Annotation Section of the DDBJ/ENA/GenBank databases under the accession numbers TPA: BK067254-BK067259: *Acentria ephemerella* BK067254 (primary allele), BK067255 (alternate allele), *Nymphula nitidulata*: BK067256 (primary allele), BK067257 (alternate allele), *Parapoynx stratiotata*: BK067258 (primary allele), and BK067259 (alternate allele). All supporting data and materials are available as supplementary material (see jkae093_Supplementary_Data). The amino acid compositions, alternate assemblies, repeat-masked assemblies, and structural (Braker proteins fasta and gtf file) and functional annotations (xml files with blasted proteins and Blast2GO reports) have been deposited at figshare under the following link: https://doi.org/10.6084/m9.figshare.24547678.v1. Supplemental material available at G3 online.

## Code availability

All bioinformatic tools for data analysis were used according to their manuals, and their versions and parameters have been introduced in the *Materials and methods* section. No custom code was used.
