# Supplementary material for: Genomic resources of aquatic Lepidoptera, Elophila obliteralis and Hyposmocoma kahamanoa, reveal similarities with Trichoptera in amino acid composition of major silk genes
Source: G3 (Bethesda). 2024 May 9;14(9):jkae093. doi: 10.1093/g3journal/jkae093 (PMC11373647; doi:10.1093/g3journal/jkae093)
Supplement: jkae093_Supplementary_Data [file jkae093_supplementary_data.pdf]

Supplementary Material Notes, Figures and Tables

Content

Supplementary Note S1 *Hyposmocoma kahamanoa* partial h-fibroin sequences
Figure S1 Taxon-annotated GC-coverage (TAGC) plots of *Elophila obliteralis*
Figure S2 Taxon-annotated GC-coverage (TAGC) plots of Hyposmocoma kahamanoa
Figure S3 Genomescope2 profiles for *Elophila obliteralis*
Figure S4 Coverage distribution per position and genome size estimate from backmap.pl for *Elophila obliteralis*
Figure S5: Genomescope2 profiles for *Hyposmocoma kahamanoa*
Figure S6 Coverage distribution per position and genome size estimate from backmap.pl for *Hyposmocoma kahamanoa*
Supplementary Note 2 Phylogenetic analyses of terminal regions of the h-fiborin
Figure S7 Alignment of n-terminal regions of h-fibroin sequences of Trichoptera and terrestrial/ aquatic Lepidoptera
Figure S8 Consensus tree of maximum likelihood analysis of n-terminal h-fibroin regions
Figure S9 Alignment of c-terminal regions of h-fibroin sequences of Trichoptera and terrestrial/ aquatic Lepidoptera
Figure S10 Consensus tree of maximum likelihood analysis of n-terminal h-fibroin regions
Figure S11 Schematic representation of the h-fibroin of *Acentria ephemerella*
Figure S12 Schematic representation of the h-fibroin of *Nymphula nitidulata*
Figure S13 Schematic representation of the h-fibroin of *Parapoynx stratiotata*
Figure S14 Amino acid composition of full-length h-fibroin of *Acentria ephemerella*
Figure S15 Amino acid composition of full-length h-fibroin of *Elophila obliteralis*
Figure S16 Amino acid compostion of partial h-fibroin of *Hyposmocoma kahamanoa*
Figure S17 Amino acid compostion of full-length h-fibroin of *Nymphula nitidulata*
Figure S18 Amino acid compostion of full-length h-fibroin of *Parapoynx stratiotata*
Table S1: Repeat classes of both genome assemblies
Table S2: Extracted H-fibroins
Table S3: Descriptive statistics for BRAKER annotations

Supplementary Note S1: *Hyposmocoma kahamanoa* partial h-fibroin sequences

Partial n-term

DAYIQDVHRLEAMMTKTDMSSTDEYEVDQNGTVYERSTTRKKFERDGDRPTGGISGEDKIRRTFVIETDAYGHETIYE
EDVVIKKVPGKHGSSKASSAGAAGAAGAAGAAGAASAASAASAAGAAGAAGAAGAAGAAGAGSGRGLYG
RYGSYGSGSSAGAAAAAAAAADAEAAAAAAAAAAAAGAGSGLGRRGLYGSYGPGSSAGAAAAAAAAADAEAAAAAA
AAAAAAGAGSGLGRRGLYGPYGPGSSAGVAAAGAAGAAGAAGAAGAAGAAGAAGAASAAGAAGAAGAA
GAGRGLYGRYGSGSSAGAAAAAAAAANAEAAAAAAAAAAAAAGAGSGLGSRGLYGLYGPGSSAGAAAAAAAAADAEA
AAAAAAAAAAAGAGSGLGRRGLYGSYGPGSSAGAAAAGAAGATGAEYSALIPLL

Partial c-term

SGSGSGGRGSYGSGSSAGAAAAAAAAAAAEADAAAAAAAAAGAGSGLGRGGLYGSYGPGSSAGVAAAAAAADAE
AAAAAAAAAAAAGAAGAAGAAGAGAAGAGAAGAGSSSTYAPYGWNGVRKACRLTRRQFSVKIGTRRQPCTTC

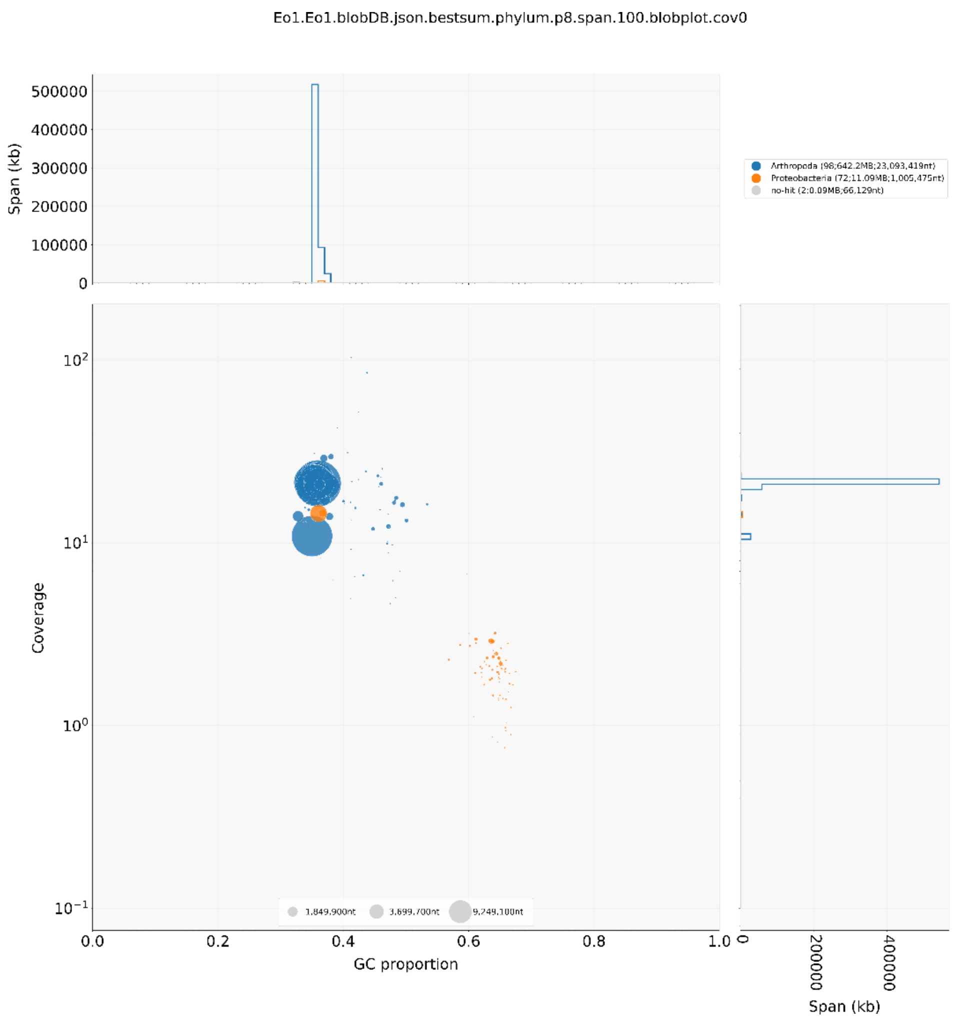

Figure S1 Taxon-annotated GC-coverage (TAGC) plots of *Elophila obliteralis.* Circles indicate contigs and the color indicates the best match to taxon annotation. The upper and right-hand panel show the total span of contigs (kb) given GC proportion***.*** We removed contigs not assigned to Arthropoda that were not in the range of the coverage (4.6591-103.4052) and GC content (0.3314-0.5401) of the Arthropod-assigned contigs.

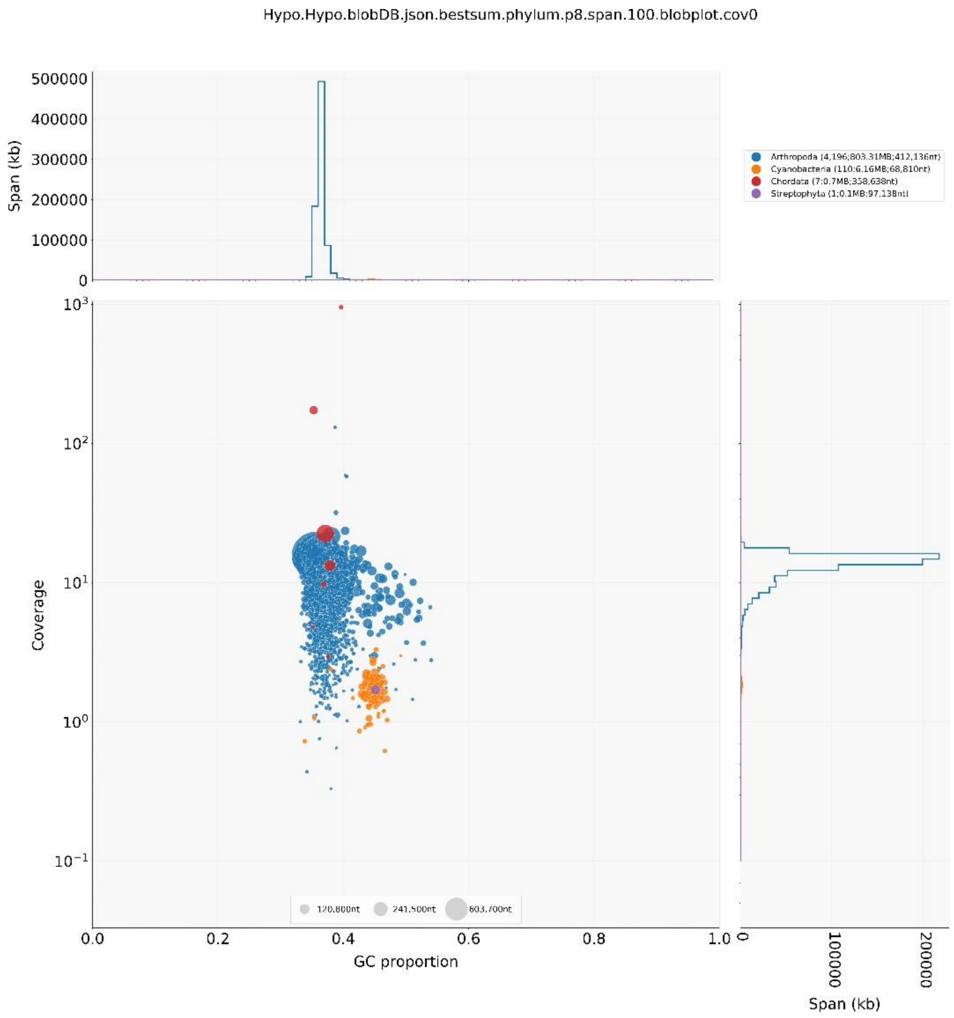

Figure S2 Taxon-annotated GC-coverage (TAGC) plots of Hyposmocoma kahamanoa. Circles indicate contigs and the color indicates the best match to taxon annotation. The upper and right hand panel show the total span of contigs (kb) given GC proportion**.** We removed contigs not assigned to Arthropoda that were not in the range of the coverage (0.3329-131.0185) and GC content (0.3278-0.5977) of the Arthropod assigned contigs.

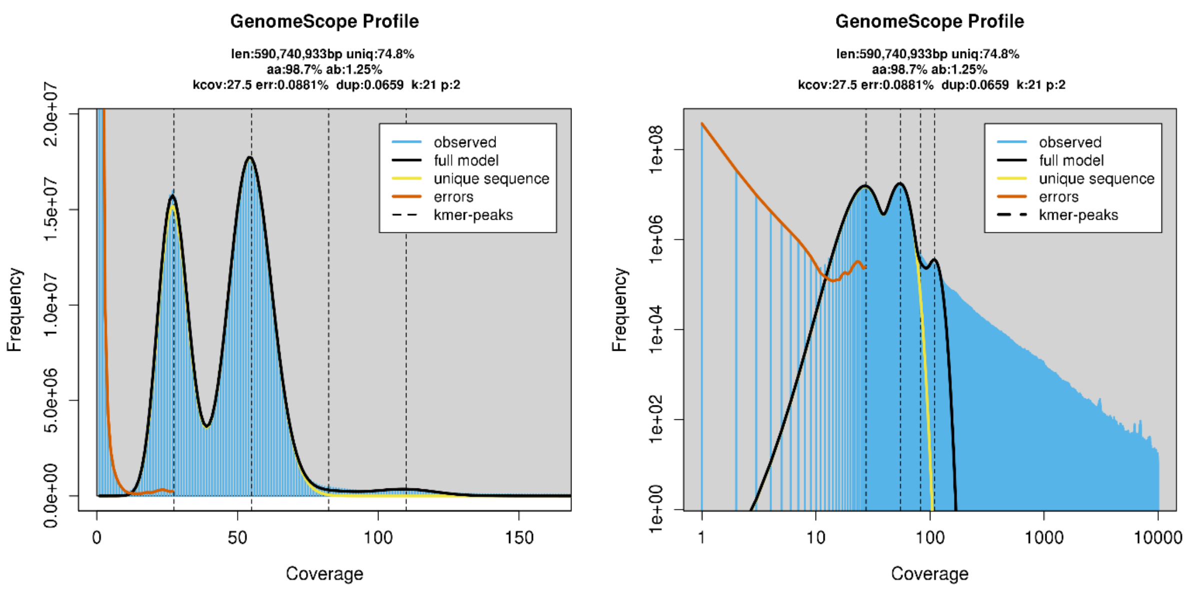

Figure S3 Genomescope2 profiles for *Elophila obliteralis.* **Left** linear plot **Right** log plot; len: inferred total genome length, uniq: percent of the genome that is unique (not repetitive), kcov: mean *k-mer* coverage for heterozygous bases, err: error rate of the reads, dup: average rate of read duplications.

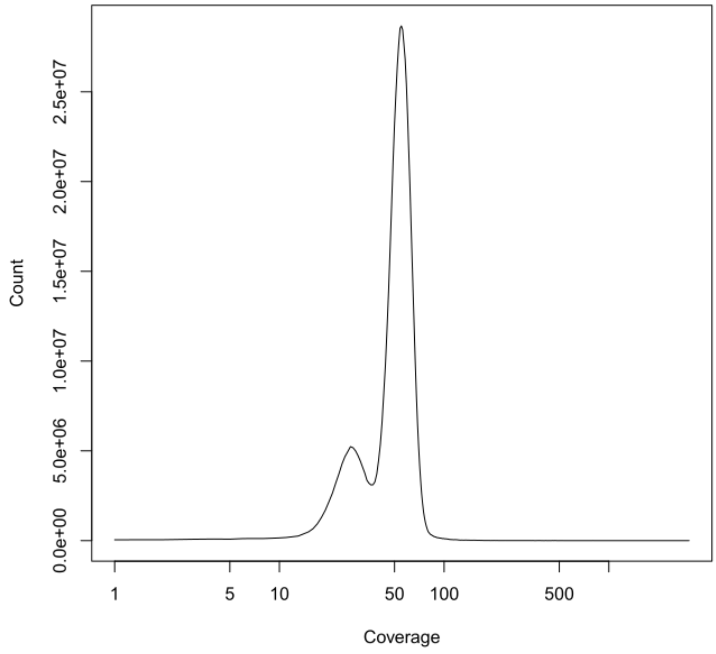

Figure S4 Coverage distribution per position and genome size estimate from backmap.pl for *Elophila obliteralis.* The x-axis is given in log-scale. Coverage distribution per position. The x-axis is given in log-scale. Mapped nucleotides: 34.56 Gb. The peak coverage is 55. This results in genome size estimation of 628.33 Mb.

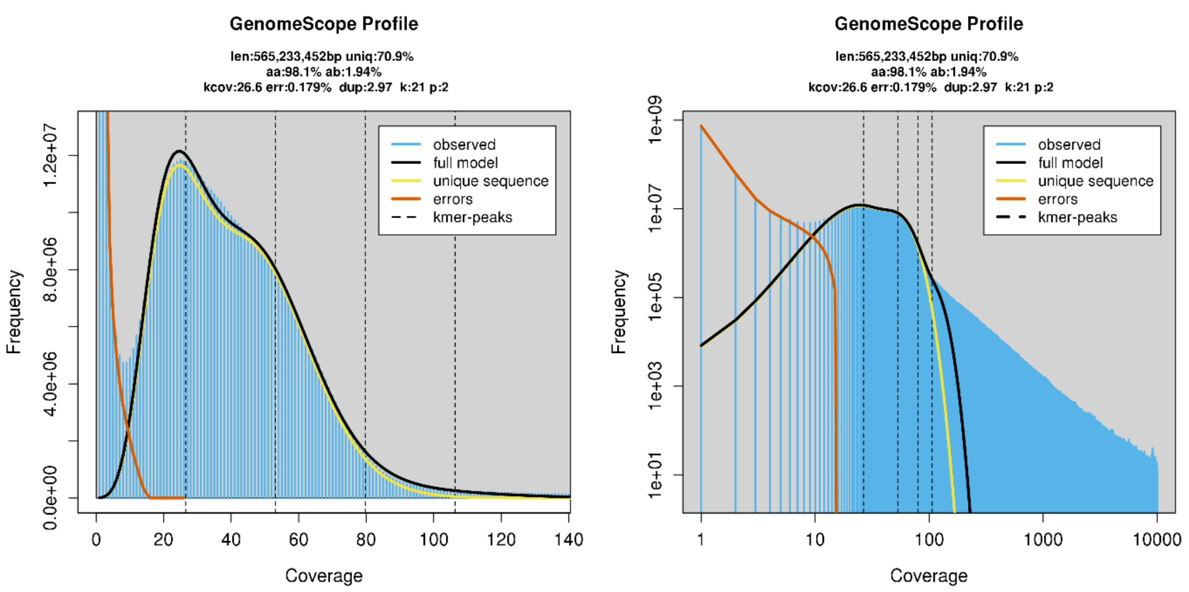

Figure S5

Figure S5: Genomescope2 profiles for *Hyposmocoma kahamanoa.* Left linear plot **Right** log plot; len: inferred total genome length, uniq: percent of the genome that is unique (not repetitive), kcov: mean *k-mer* coverage for heterozygous bases, err: error rate of the reads, dup: average rate of read duplications.

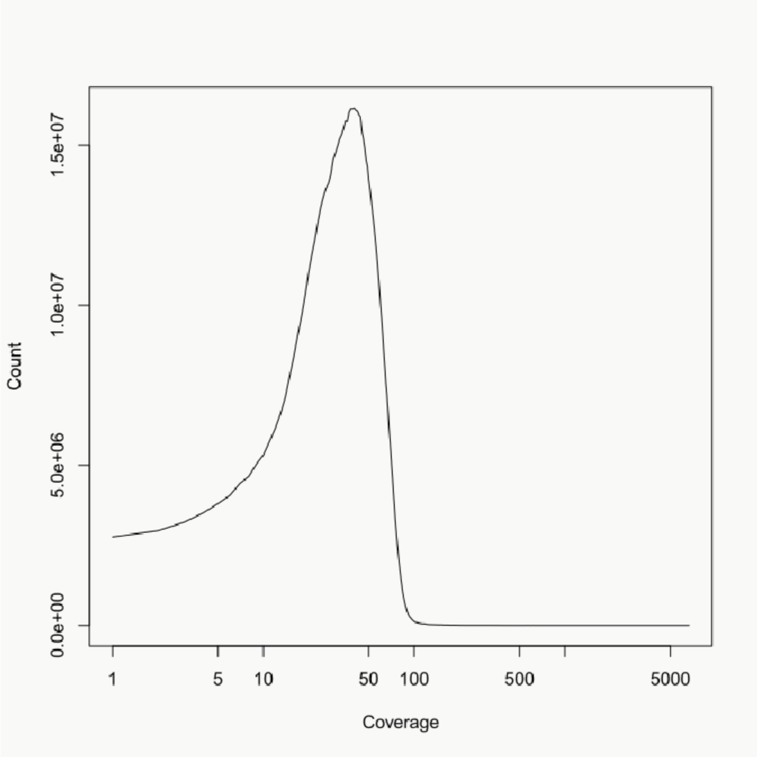

Figure S6 Coverage distribution per position and genome size estimate from backmap.pl for *Hyposmocoma kahamanoa.* The x-axis is given in log-scale. Coverage distribution per position. The x-axis is given in log-scale. Mapped nucleotides: 33.09 Gb. The peak coverage is 40. This results in genome size estimation of 827.25 Mb.

Supplementary Note 2 Phylogenetic analyses of terminal regions of the h-fiborin

Terminal regions of h-fibroins were aligned in Geneious Prime with MUSCLE 3.8.425.

Phylogenetic trees were examined with RAxML 8.2.11 with protein model GAMMA GTR and the Rapid bootstrapping algorithm with 1,000 bootstrap replicates. Consensus trees were generated with the Geneious Consensus Tree Builder with a support Threshold of 50%.

All phylogenetic trees can be found in nexus format at Figshare: https://doi.org/10.6084/m9.figshare.24547678.v1

Distance matrices with the % of bases which are identical are exported from Geneious and can be found at Figshare: https://doi.org/10.6084/m9.figshare.24547678.v1

Figure S7 Alignment of n-terminal regions of h-fibroin sequences of Trichoptera and terrestrial/ aquatic Lepidoptera

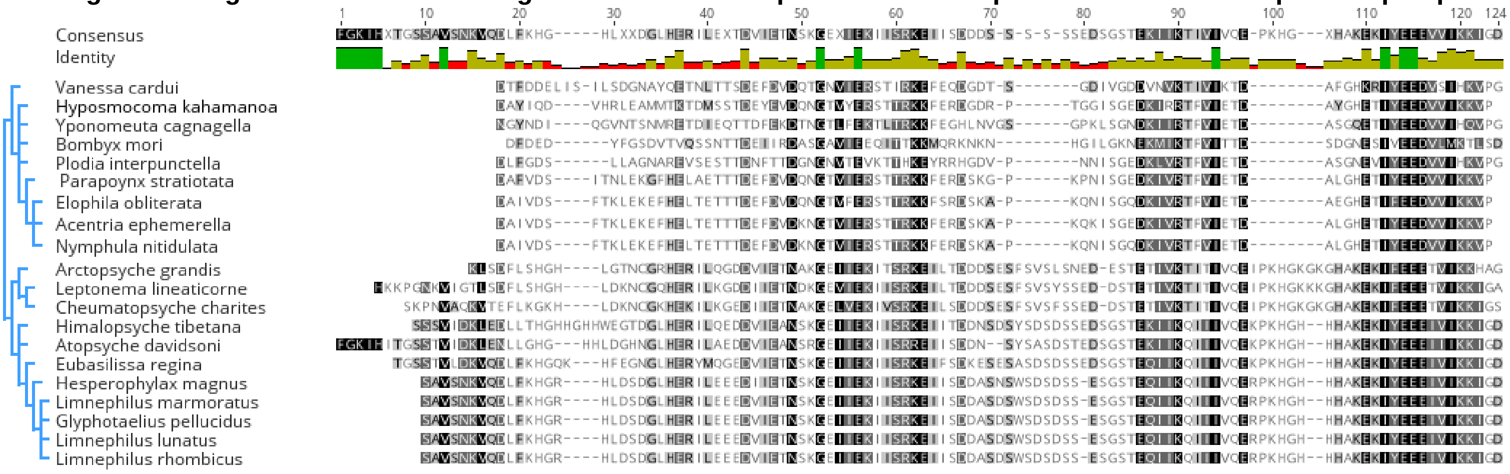

Figure S8 Consensus tree of maximum likelihood analysis of n-terminal h-fibroin regions

green= terrestrial Lepidoptera, light-blue=aquatic Lepidoptera, dark-blue=Trichoptera

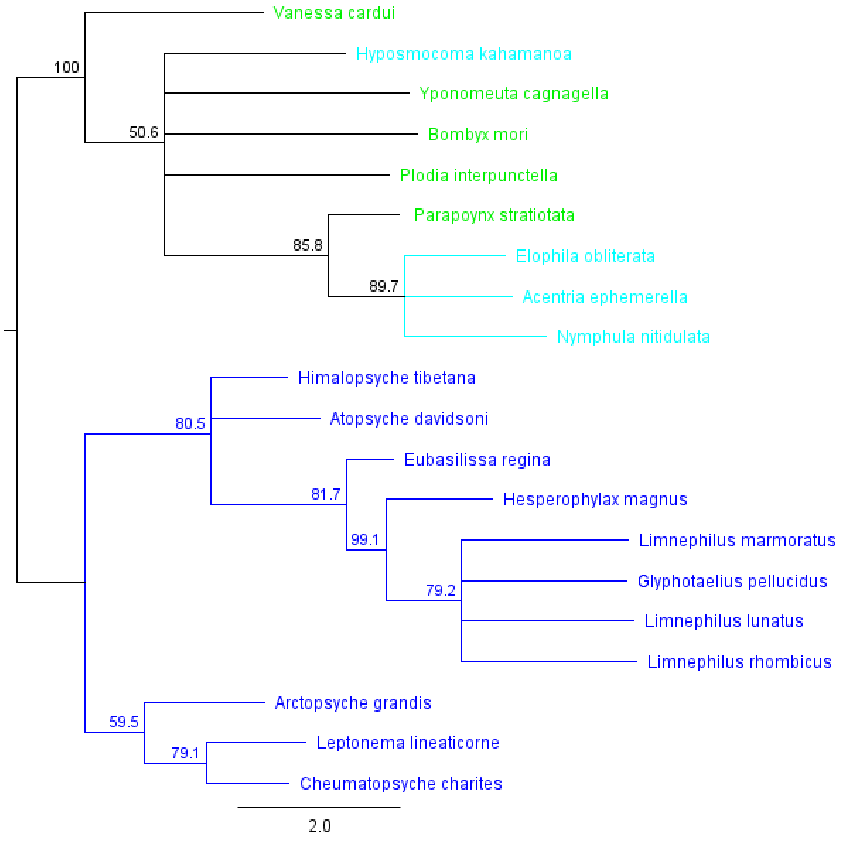

Figure S9 Alignment of c-terminal regions of h-fibroin sequences of Trichoptera and terrestrial/ aquatic Lepidoptera

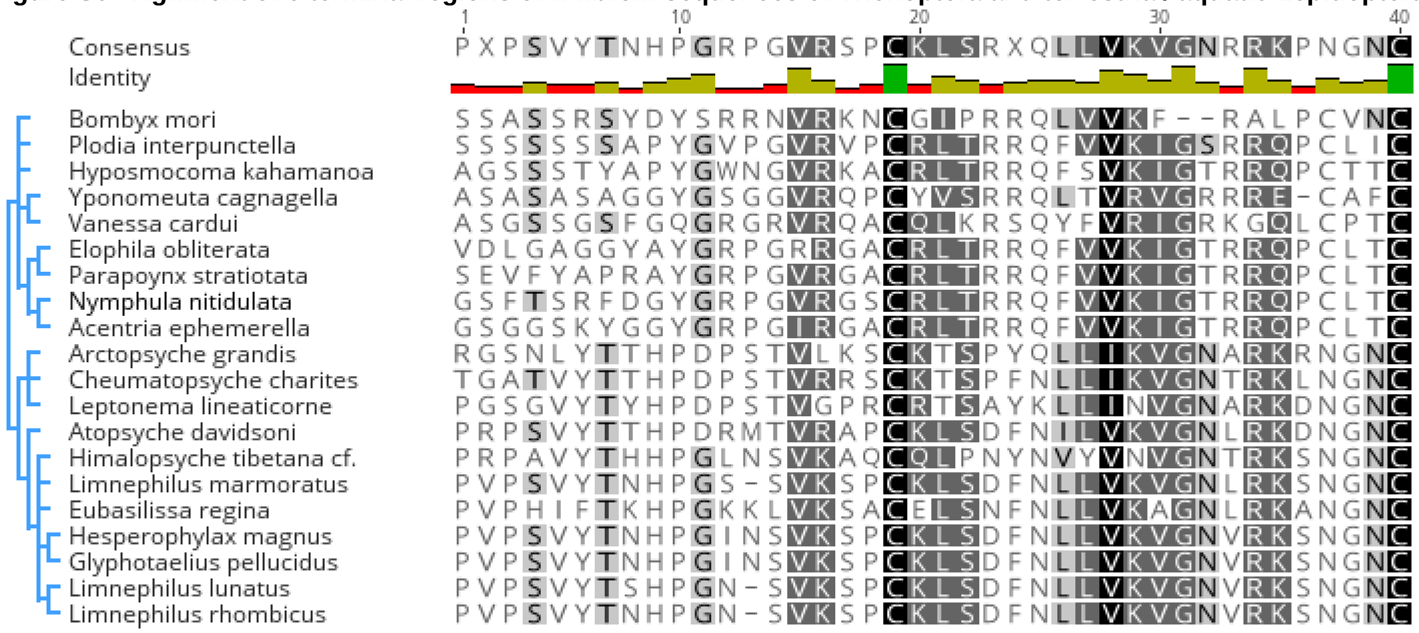

Figure S10 Consensus tree of maximum likelihood analysis of n-terminal h-fibroin regions
green= terrestrial Lepidoptera, light-blue=aquatic Lepidoptera, dark-blue=Trichoptera

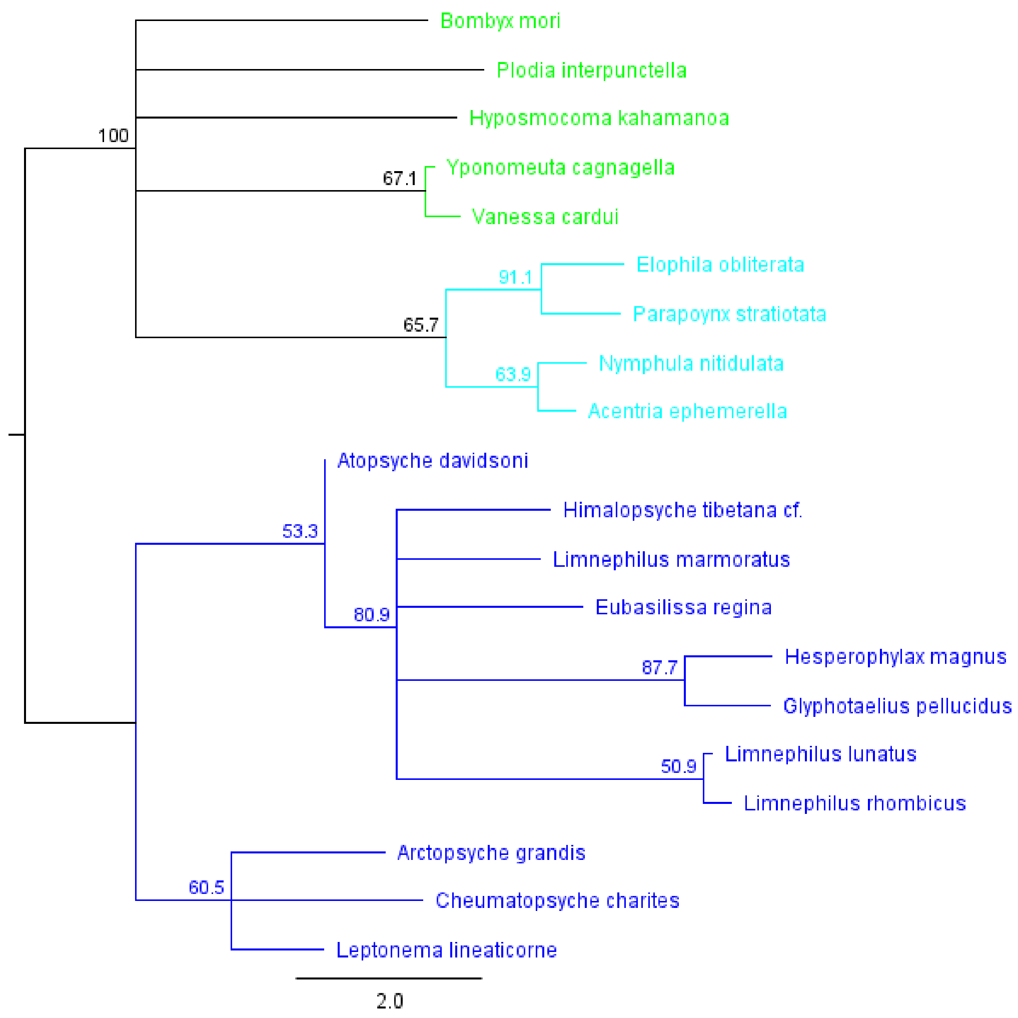

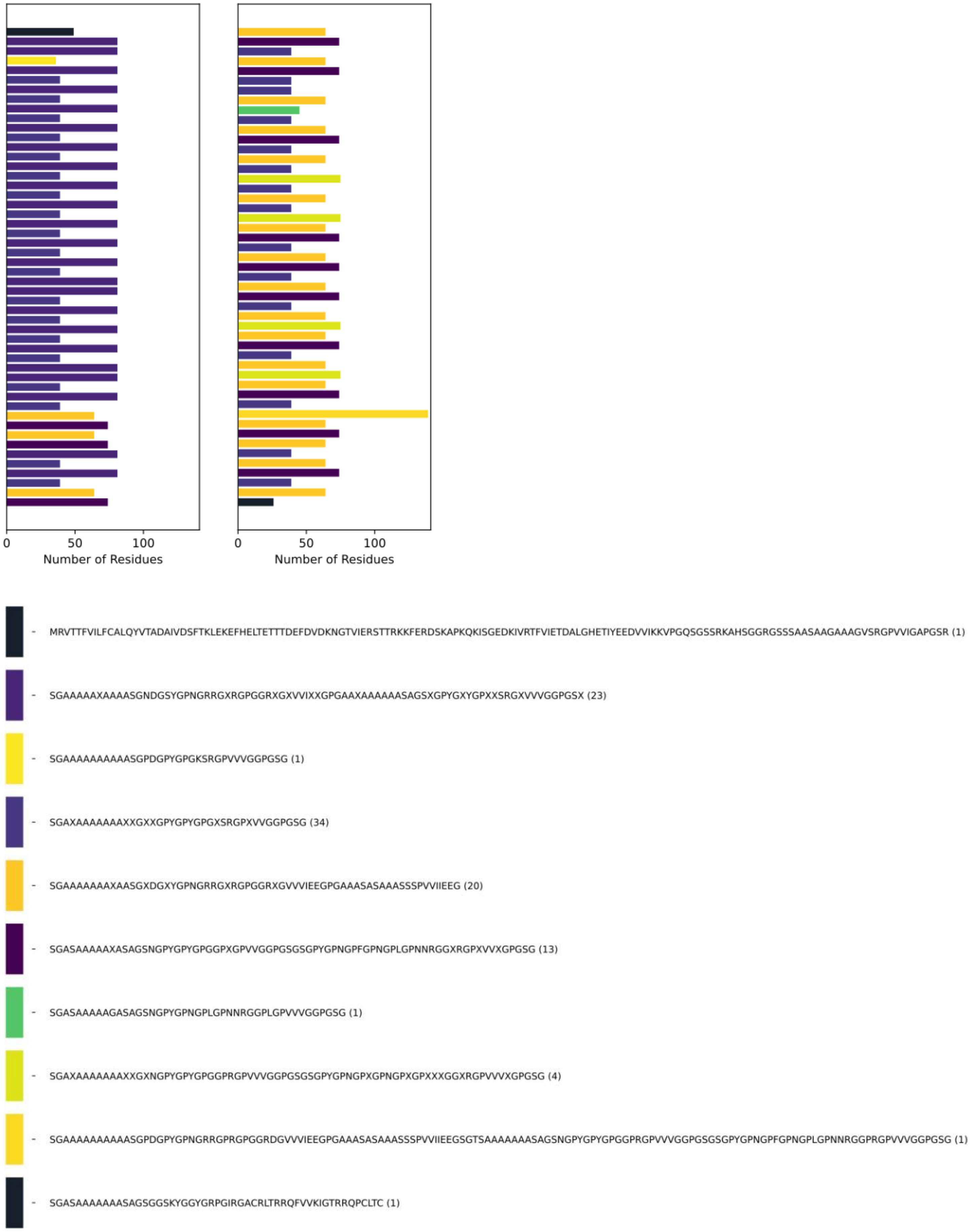

Figure S11 Schematic representation of the h-fibroin of *Acentria ephemerella*. Identity and ordering of repeat motifs are shown. Repetitive units with the *N*-terminus and transfer region at the beginning and the *C*-terminus and transfer region at the end (black, bold). The gene is split into two panels. “X” indicates a variable site. The color corresponds with the ordering of the repeats shown below. The numbers in parentheses refer to the number of times that particular motif is repeated across the gene.

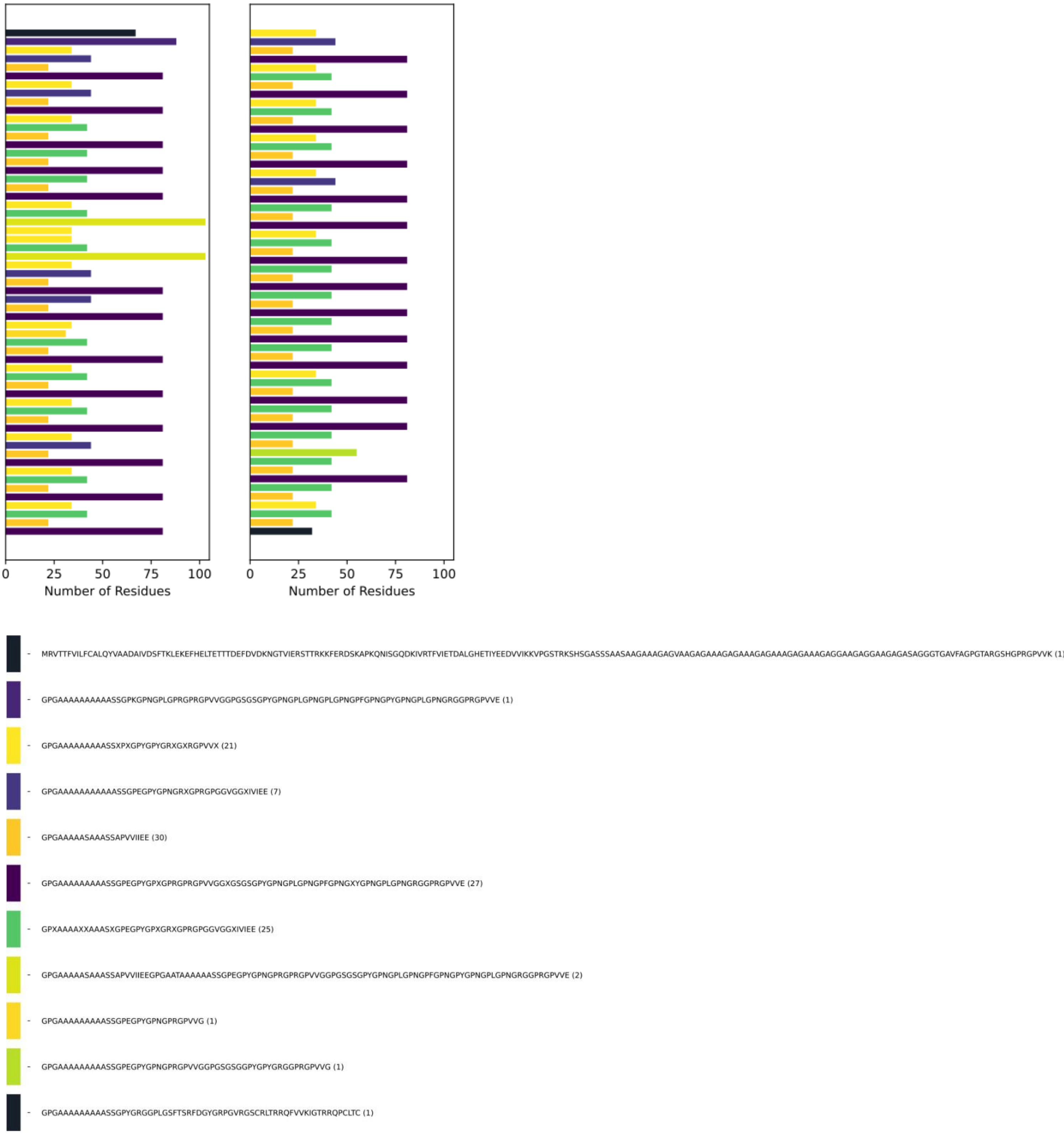

Figure S12 Schematic representation of the h-fibroin of *Nymphula nitidulata*. Identity and ordering of repeat motifs are shown. Repetitive units with the *N*-terminus and transfer region at the beginning and the *C*-terminus and transfer region at the end (black). The gene is split into two panels. "X" indicates a variable site. The color corresponds with the ordering of the repeats shown below. The numbers in parentheses refer to the number of times that particular motif is repeated across the gene.

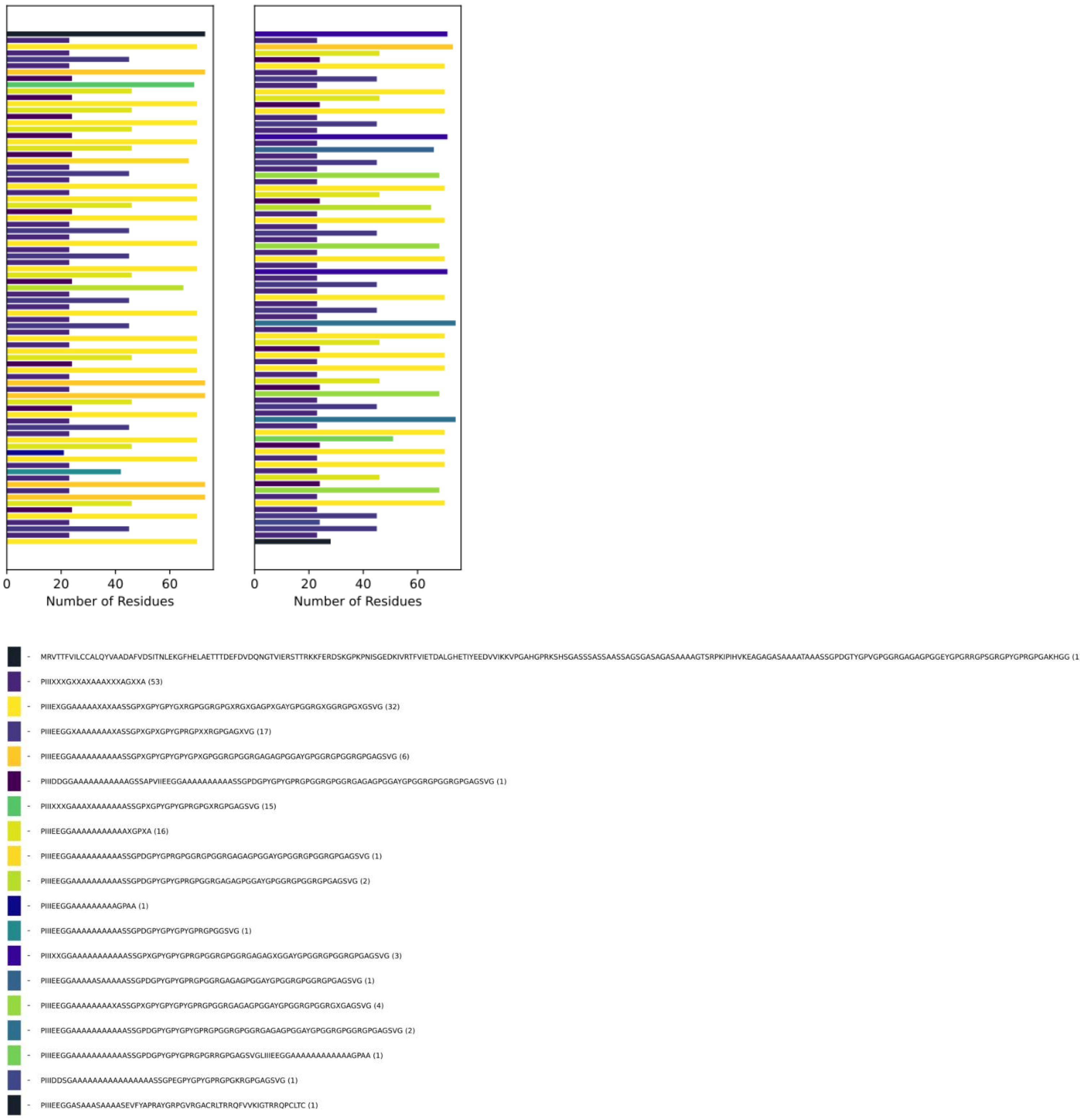

Figure S13 Schematic representation of the h-fibroin of *Parapoynx stratiotata*. Identity and ordering of repeat motifs are shown. Repetitive units with the *N*-terminus at the beginning and the *C*-terminus at the end (black). The gene is split into two panels. “X” indicates a variable site. The color corresponds with the ordering of the repeats shown on the left. The numbers in parentheses refer to the number of times that particular motif is repeated across the gene.

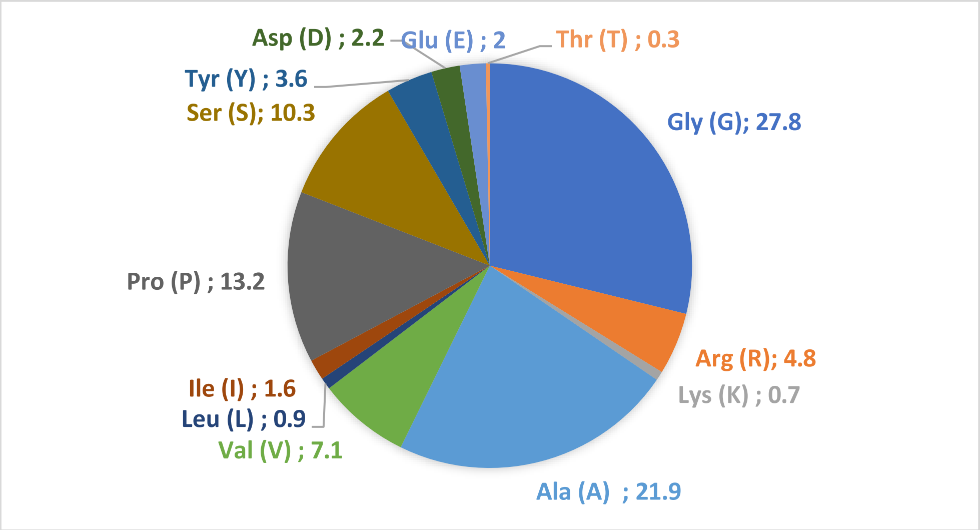

Figure S14 Amino acid composition of full-length h-fibroin of *Acentria ephemerella*

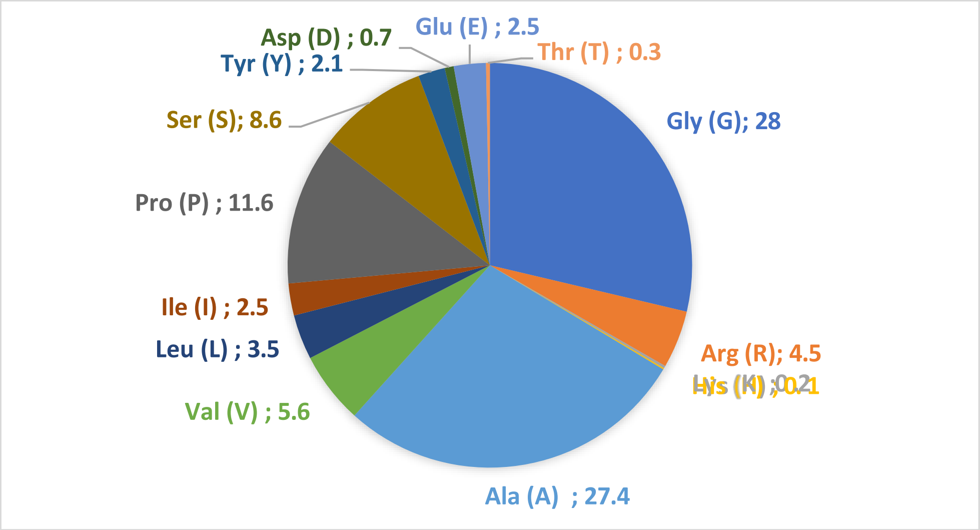

Figure S15 Amino acid composition of full-length h-fibroin of *Elophila obliteralis*

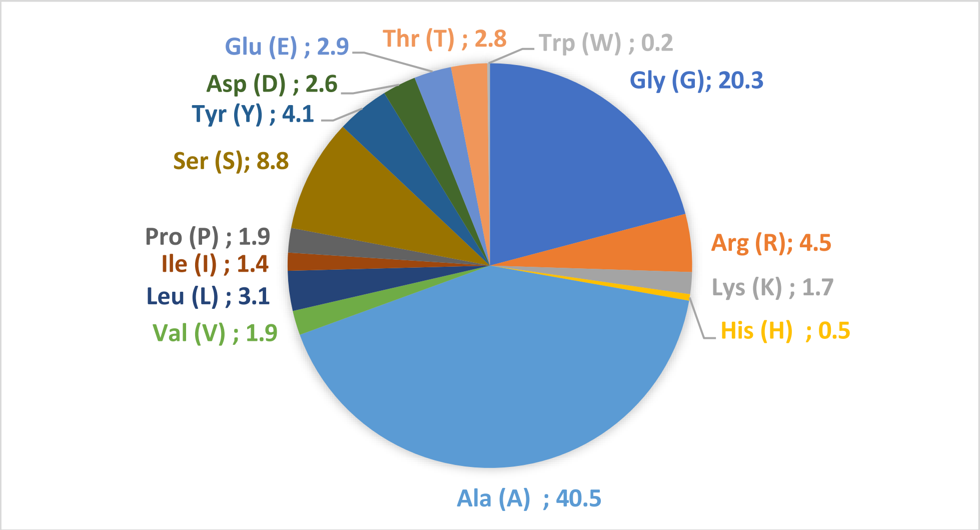

Figure S16 Amino acid compostion of partial h-fibroin of *Hyposmocoma kahamanoa*

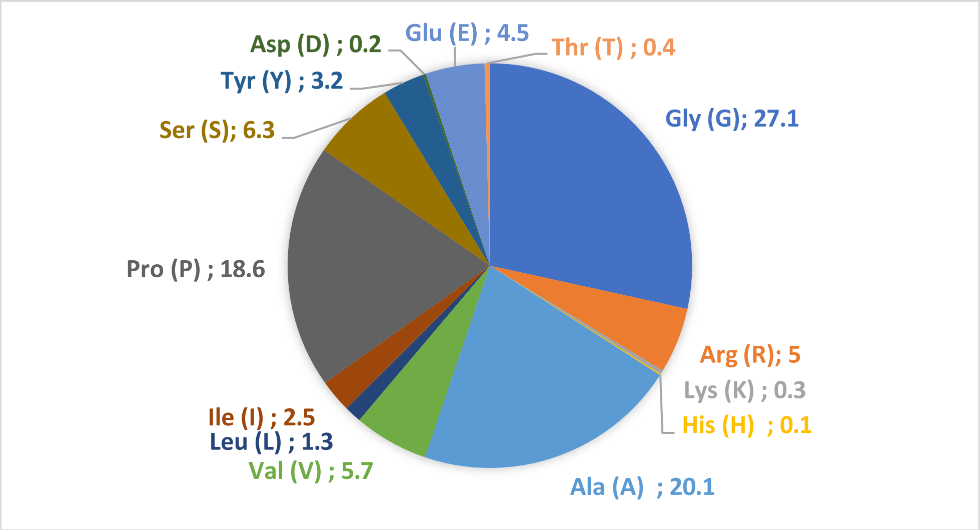

Figure S17 Amino acid compostion of full-length h-fibroin of *Nymphula nitidulata*

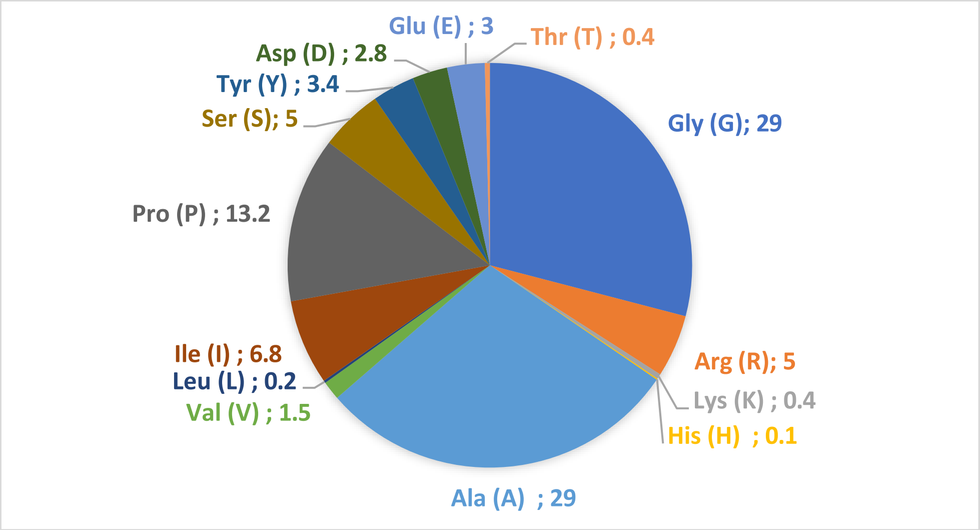

Figure S18 Amino acid compostion of full-length h-fibroin of *Parapoynx stratiotata*

Table S1: Repeat classes of both genome assemblies

**Repeats (% of sequence)**	***Elophila obliteralis***	***Hyposmocoma kahamanoa***
Retroelements	24.16	24.77
SINES	7.49	7.41
LINES	14.57	15.20
LTR elements	*2.10*	2.16
DNA transposons	2.82	5.95
Rolling-circles	3.76	1.8
Unclassified repeats	20.94	25.36
Total interspersed repeats	47.91	56.09
Small RNA	3.85	3.78
Satellites	0.0	0.3
Simple repeats	0.83	0.74
Low complexity	0.1	0.10

Table S2: Extracted H-fibroins

	Haplotype	NCBI Genbank Accession	Identified in	Location in genome
Acentria ephemerella	primary	2741941	GCA_943193645.1	OW971889.1:8,064,226-8,083,676
	alternate	2741956	GCA_943193655.1	CALPDL010000018.1:7,273,045-7,293,512
Elophila obliteralis	primary	OR533279	This study: JAVLVO000000000	ptg00011l: 6,524,985-6,506,906 (reverse)
	alternate	OR533280	This study: elophila_e01_hifiasm_hap2.p_ctg.fa	h2tg00014l:13,233,959-14,253,506
Nymphula nitidulata	primary	2741981	GCA_947347705.1	OX374635.1:5,983,305-5,965,612(rev)
	alternate	2741995	GCA_947347715.1	CANAFF010000310.1:186,637-206,343
Parapoynx stratiotata	primary	2742005	GCA_910589355.1	OU342475.1:10,953,369-10,975,363
	alternate	2742006	GCA_910589245.1	CAJUUE010000100.1:214,022-192,547(rev)

Table S3: Descriptive statistics for BRAKER annotations

	Elophila obliteralis	***Hyposmocoma kahamanoa***
**Number of proteins**	21,179	35,668
**% BUSCO (n=2124)**	C:95.9%[S:88.3%,D:7.6%],F:0.9%,M:3.2%	C:72.3%[S:66.4%,D:5.9%],F:12.4%,M:15.3%
**Number of transcripts**	19,952	33,517
**Largest number of exons in all transcripts**	133	95
**Monoexonic transcripts**	5,469	10,768
**Multiexonic transcripts**	14,483	22,749
**Mono:Mult Ratio:**	0.38	0.47
